# Corrections in Grasp Posture in Response to Modifications of Action Goals

**DOI:** 10.1371/journal.pone.0043015

**Published:** 2012-09-06

**Authors:** Charmayne M. L. Hughes, Christian Seegelke, Marnie Ann Spiegel, Corinna Oehmichen, Julia Hammes, Thomas Schack

**Affiliations:** 1 Neurocognition and Action Research Group, Faculty of Psychology and Sport Sciences, Bielefeld University, Bielefeld, Germany; 2 Research Institute for Cognition and Robotics (CoR-Lab), Bielefeld, Germany; 3 Center of Excellence Cognitive Interaction Technology (CITEC), Bielefeld, Germany; 4 Institute of Movement Science, Department of Sport and Health Science, Technical University of Munich, Munich, Germany; Weill Cornell Medical College, United States of America

## Abstract

There is ample evidence that people plan their movements to ensure comfortable final grasp postures at the end of a movement. The end-state comfort effect has been found to be a robust constraint during unimanual movements, and leads to the inference that goal-postures are represented and planned prior to movement initiation. The purpose of this study was to examine whether individuals make appropriate corrections to ensure comfortable final goal postures when faced with an unexpected change in action goal. Participants reached for a horizontal cylinder and placed the left or right end of the object into the target disk. As soon as the participant began to move, a secondary stimuli was triggered, which indicated whether the intended action goal had changed or not. Confirming previous research, participants selected initial grasp postures that ensured end-state comfort during non-perturbed trials. In addition, participants made appropriate on-line corrections to their reach-to-grasp movements to ensure end-state comfort during perturbed trials. Corrections in grasp posture occurred early or late in the reach-to-grasp phase. The results indicate that individuals plan their movements to afford comfort at the end of the movement, and that grasp posture planning is controlled via both feedforward and feedback mechanisms.

## Introduction

Many of our activities of daily living require that we physically interact with one or more objects. These object manipulations are typically influenced by the physical properties of the object [1]–[3], the affordances provided by the object [4], and our intentions [2]. There is also ample evidence suggesting that the action goals of a task are an important determinant in the planning and execution of the motor sequence subtending reach-to-grasp movements [2], [5]–[8]. For example, Marteniuk et al. [2] compared movement trajectories of the hand during a grasping and throwing task to hand trajectories during a grasping and fitting task. Participants took longer to reach for the object, and spent more time in the decelerative portion of the reach-to-grasp phase when the object was to be fitted into a hole (high degree of precision) than when they had to throw it into a bucket (low degree of precision).

Similarly, in Ansuini et al. [6] participants reached for a plastic bottle filled with water, and then 1) grasped the bottle without performing any subsequent action (grasp), 2) grasped the bottle, lifted, and then threw the bottle into a cardboard container (lift), 3) grasped, lifted and then placed the bottle on a target (place), 4) grasped, lifted, and then poured the water from the bottle into a container (pour), or 5) grasped, lifted, and then passed the bottle to the experimenter (pass). Ansuini et al. [6] found a gradual shortening of reach duration for the “pour”, “place”, “pass”, and “throw” conditions, respectively, which likely relates to the accuracy demands associated with the “pour” and “place” conditions, compared to the “pass” and “throw” conditions. The authors also observed greater middle and ring finger extension in the “pour”, compared to all other conditions, and larger index-middle and middle-ringer abduction angles for the “throw” than for all other conditions. In sum, these results indicate that the action end-goals of a task strongly influence the planning and execution of hand and finger movements.

The influence of action end-goals on motor planning and execution has also been observed on a more macroscopic level of behavior. In his now seminal bar transport experiment, Rosenbaum et al. [8] asked participants to grasp a horizontally positioned bar and place it in a vertical position to either a left or a right target. When the left side of the bar was to be placed to either the left or right target, all participants grasped the bar with an underhand grip. However, when the right side of the bar was to be placed to either target, participants always grasped the bar with an overhand grip. Thus, regardless of target location, participants grasped the object so that the hand ended in a comfortable posture. The sensitivity toward comfortable (and more controllable) final goal postures is called the end-state comfort effect. Since the original exposition by Rosenbaum et al. [8], subsequent research has provided evidence that comfortable goal postures are represented and planned prior to movement initiation [9].

However, there are instances when we must make rapid adjustments in our behavior due to unexpected changes in the environment. One technique by which researchers can examine compensatory motor control mechanisms is to induce a perceptual or physical change the shape and size of an object [10]–[13], or the location of the target [14]. For example, in Paulignan et al. [14], participants were asked to reach, grasp, and lift one of three possible dowels. In 20% of trials, the light was unexpectedly shifted from the center dowel to either the left or to the right dowel as soon as subject initiated their reaching movements. Paulignan et al. [14] found that perturbing the target location had considerable effects on reach-to-grasp kinematics. Although the initial portion of the movement were similar for non-perturbed and perturbed trials, after approximately 275 ms the spatial trajectories of perturbed trials curved towards the new target location. These adjustments in the spatial trajectory were accompanied by lower peak velocity and earlier time to peak velocity values in perturbed, compared to non-perturbed trials.

In sum, results of studies using perturbation paradigms have reported that participants make appropriate on-line adjustments in hand shape and trajectory when faced with a sudden change in the environment. These studies also report that the consequences of on-line arm trajectory modifications include overall increases in movement time, relative lengthening of the deceleration phase, and bimodal grip aperture profiles [11], [14]–[17]. In the aforementioned perturbation studies individuals must make corrective adjustments in hand shape and trajectory in order to successfully accomplish the new action goal (i.e., grasp the displaced object).

In the current experiment, we investigated whether participants would adjust their reach-to-grasp movements when perturbations in action goal do not necessarily warrant the need for compensatory motor control mechanisms. To address this issue, we modified the bar transport task of Rosenbaum et al [8] and introduced a perturbation in action goal at reach-to-grasp onset. Participants were required to reach for a horizontal cylinder and place either the left or right end of the object into the target disk. When participants initiated their reach-to-grasp movement, the secondary stimuli was triggered, which served to indicate whether the required object end orientation had changed (perturbed trials = 20%) or not (non-perturbed trials = 80%).

The question of primary interest is whether individuals would make appropriate corrections to ensure comfortable final goal postures when faced with an unexpected change in action goal. If end-state comfort is a highly prioritized planning constraint, then it follows that participants should correct their reach-to-grasp movements to ensure comfortable end-states when an unexpected change in action goal occurs. This interpretation would be supported if similar end-state comfort values were found for non-perturbed and perturbed trials. However, given that the object can be placed into the target disc using a comfortable or an uncomfortable final grasp posture, participants do not necessarily need to modify their grasp postures in response to action goal perturbation. If this is the case, end-state comfort compliance should be significantly lower for perturbed, compared to non-perturbed trials.

In this paper we also provide a complete kinematic description of unimanual grasping and placing during the bar transport task in non-perturbed conditions, and compare the data from non-perturbed trials to conditions in which the action goal is unexpectedly changed. If participants modify their reach-to-grasp movements to ensure end-state comfort, we expect that the change in action goal would disturb the organization of hand shaping and kinematics (increased movement time, with bimodal grasp aperture profiles) of the reach-to-grasp phase of the movement.

## Methods

### Participants

Sixteen students from Bielefeld University were recruited to take part in this experiment in exchange for 5€. The dataset from one participant was removed due to equipment malfunction, leaving us with a sample of fifteen participants (mean age = 24.7, SD = 2.05, 9 men and 6 women). All participants were right handed, as determined by the Revised Edinburgh Handedness Inventory [18]. Participants reported normal or corrected to normal vision, and did not have any known neuromuscular disorders. The methodology and written consent form for this study were approved by the ethics committee at Bielefeld University, and conformed to the declaration of Helsinki. All participants gave their informed written consent to participate in the study.

### Apparatus & stimuli

The experimental apparatus is shown in Figure 1. The set-up was positioned on a height adjustable shelf. The manipulated object was a grey PVC cylinder (20 cm in height, 6 cm in diameter, 784 g in weight) that had a band of blue electrical tape (2 cm) wrapped around one end, and a band of yellow electrical tape (2 cm) wrapped around the other end. The object was horizontally positioned on the cradle (20 cm length) that held the object 20.5 cm above the table. The start orientation of the object was kept constant for each participant across all trials (e.g., blue end always pointing to the left or to the right). The start orientation of the object was counterbalanced across participants, so that half of the participants performed the task when the object was placed with the blue end of the cylinder pointing to the left, and the other half performed the task with the blue end of the cylinder pointing to the right.

**Figure 1:**
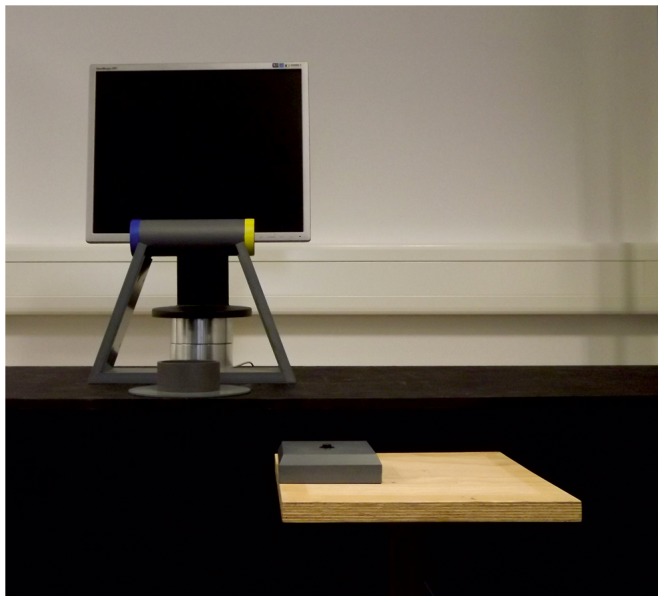
Front view of the experimental set-up.

The target was a PVC hollow cylinder (4 cm in depth, 8 cm in diameter) located 10 cm in front of the object cradle. Located to the right of the participant was a start button (10 cm×10 cm×10 cm) placed on a height adjustable table, which was used to collect reaction time values during the experiment. Pressure-sensitive micro switches were embedded within the start button, object cradle, and target disc, and were used to collect reaction time, reach-to-grasp time, and grasp-to-place time.

A 43 cm flat screen monitor (Sync Master 943T, Samsung) was placed 15 cm behind the object cradle and delivered visual stimuli throughout the experiment. The visual stimuli consisted of a representation of the required end orientation of the object. The auditory stimulus consisted of either a low (400 Hz) or high (750 Hz) tone. The auditory stimulus was presented at 60 dB (500 ms duration) through two loudspeakers (Logitech Z 323 2.1-CH PC, Logitech) situated 15 cm to either side of the computer monitor (45° and 135° in azimuth). The secondary (final) stimuli consisted of a visual object representation and a tone, which was presented simultaneously. In non-perturbed trials, the secondary visual stimulus was identical to the initial stimulus (e.g., initial stimulus left end-down and secondary stimulus left end-down), and the auditory cue indicated that the intended action goal had not changed. In perturbed trials, the secondary visual stimulus differed from the initial stimulus (e.g., initial stimulus left end-down and secondary stimulus right end-down), and the auditory cue indicated that the intended action goal had changed. For half of the participants, the high tone (750 Hz) was associated with non-perturbed trials, and the low tone (400 Hz) was associated with perturbed trials. For the other participants, the auditory mapping was reversed. The auditory mapping was counterbalanced across participants and was known prior to the experiment. Stimulus presentation was controlled via Presentation® (Neurobehavioral Systems).

To collect kinematic data, retro reflective markers (14 mm diameter) were placed dorsally on the distal end of the third metacarpal (MCP), styloid process of the ulna (US), styloid process of the radius (RS), the thumb nail (TB), and the index finger nail (IDX) of the right hand. Data was recorded using an optical motion capture system (VICON Motion Systems, Oxford, U.K.), consisting of ten Bonita cameras with a temporal and spatial resolution of 200 Hz and 1 mm, respectively. Each trial was recorded using a Basler Pilot DV camera (Basler AG, Ahrensburg, Germany) that was used to record grasp postures. The VICON motion capture system was synchronized with both the digital video camera and stimulus presentation system.

### Procedure

After reading and filling out the written informed consent and handedness inventory forms, participants' waist and navel height were measured, and retro reflective markers were placed on the right hand. The shelf and the response button height were then adjusted to navel and waist height, respectively. Participants stood in front of the experimental setup so that the body midline coincided with the object cradle and target disc.

The temporal sequence for the experimental paradigm during non-perturbed and perturbed trials is depicted in Figure 2A and 2B, respectively. At the start of each trial, the message “Put your hand on the start key!” was displayed (in German) on the computer monitor, which prompted participants to close the thumb and index finger and push down on the start button with the side of their palm. After the start button was depressed, a fixation cross was presented for 500 ms, and after a random interval (500–1500 ms) the initial stimulus appeared on the screen. In response to the visual stimulus, participants were required to lift their hand from the start button, grasp the object from the cradle, and place the object on the target. The secondary stimulus was triggered when the participant lifted their hand from the start button. The stimulus indicated whether the required object end orientation had changed (perturbed trial) or not (non-perturbed trial).

**Figure 2:**
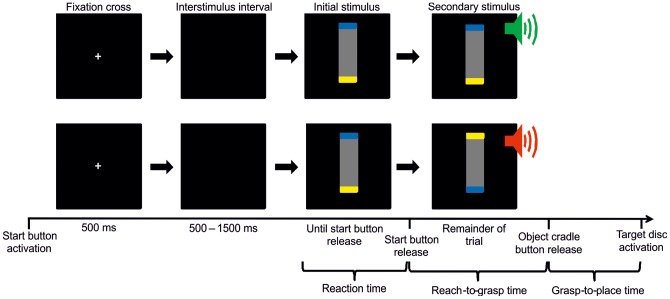
The temporal sequence of the experimental paradigm. At the beginning of each trial, a fixation cross was presented (500 ms). After a randomized interstimulus interval (500–1500 ms), the initial stimulus appeared and indicated which end of the object was to be placed on the target (left end-down, right end-down). After the participant lifted their hand from the start button, a secondary stimulus was triggered. (A) During non-perturbed trials, the visual stimulus was identical to the initial stimulus, and was paired with an auditory cue indicating that the intended object end-orientation had not changed. (B) During perturbed trials, the secondary visual stimulus differed from the initial stimulus, and was paired with an auditory cue indicating that the intended object end-orientation had changed.

Participants performed the task with their right hand, and were instructed to grasp the object with a full grip, using either an overhand or underhand posture. We emphasized speed of responding and the requirement that the final placement of the object in the target disk should replicate what was displayed on the computer monitor.

Each experimental session began with a series of acclimatization trials (15 non-perturbed movements to each object end-orientation). These trials served to familiarize the participant with the auditory mapping and the general task procedures. The order of presentation was randomized. Following the acclimatization trials, participants took a two minute rest break, and then performed 100 experimental trials. The experimental trials were divided into two blocks of 50 trials, separated by a two minute rest period. To reduce expectancy effects, the ratio of perturbed to non-perturbed trials was set to 1∶4. Thus, within each block, participants performed 20 non-perturbed trials and 5 perturbed trials to each final object end-orientation (left end-down, right end-down). All trials were fully randomized. The entire experiment lasted approximately 50 minutes.

### Data Processing

Before data analysis, we excluded trials performed in a non-instructed manner (moving prior to stimulus presentation, placing the wrong end of the object on the target) and RTs that were less than 200 ms (anticipation errors) or exceeded 800 ms (delay errors). Error trials comprised less than 3.6% of the data, and were approximately equally distributed across condition and participants. Given the low error rate, mean substitution was used to replace missing values.

Following data collection, the 3D coordinates of the retro-reflective markers were reconstructed and labeled for each individual trial. Any missing data (less than 10 frames) were interpolated using a cubic spline, and filtered using a Woltring filter [19] with a predicted mean square error value of 5 mm^2^ (Vicon Nexus 1.6). The Woltring filter is a squintic cubic spline routine and commonly used in the analysis of motion data. It is equivalent to a double Butterworth filter.

Kinematic variables were calculated using custom written MatLab programs (The MathWorks®, Version R2010a). For each trial, the time series was divided into the reach-to-grasp phase and the grasp-to-place phase. The reach-to-grasp phase was defined as the time period between when the hand left the start button to the time the object left the cradle. The grasp-to-place phase was defined as the time period between when the object left the cradle to the time the object contacted the target disc. Movement velocities for the reach-to-grasp and grasp-to-place phase were calculated using a first order central difference technique. Reach-to-grasp phase grip aperture (difference between thumb and index finger) was calculated as the Euclidean distance of TB and IDX in three-dimensional space.

The hand was modeled as a single, rigid segment. The wrist joint center (WJC) was calculated midway between the marker placed on the styloid process of the ulna and the marker placed on the styloid process of the radius. Two direction vectors were calculated, one pointing from WJC to the distal end of dorsal third metacarpal (V1 = MCP - WJC) and the second pointing from the styloid process of the radius to the styloid process of the ulna (V2 = US - RS). The hand center (HC) was defined on a plane normal to V1×(V2×V1). It was positioned palmar from MCP at a distance of (hand thickness+marker diameter)/2 in a way that MCP - HC and WJC - HC formed a right angle. A local hand coordinate system was then defined, with the origin set at WJC. The Y-axis was defined as the vector pointing from WJC to HC (Y = HC - WJC). The Z-axis was defined by the cross product of the wrist axis (US - RS) and the Y-axis. The X-axis was defined as the cross product of the Y-axis and the Z-axis) to create a right-handed coordinate system. Pro-/ supination angles were calculated as a transformation of the laboratory's coordinate system into the local hand coordinate system. The rotations were conducted in the sequence Z-X-Y around floating axes. The laboratory's coordinate system was defined with the Z-axis pointing upwards and the X- and Y-axis parallel to the floor, such that the rotational axis for pro-supination of the hand was aligned with the Y-axis of the hand and the pro-/ supination angle was zero when the hand was parallel to the floor in a palm-down orientation. Pronation of the hand resulted in a decrease of the hand pro-/ supination angle, whereas hand supination resulted in an increase of the hand pro-/ supination angle.

### Data Analysis

To investigate whether unexpected changes in action goal affect the organization of grasp posture planning we compared end-state comfort satisfaction between conditions in which the intended action goal had changed (perturbed) with those in which the intended action goal had not changed (non-perturbed). In accordance with previous studies using a similar experimental set up [8], end-state comfort satisfaction was defined by initial grasps that resulted in thumb up postures at the end of the movement. Thus, for final left end-down trials, end-state comfort was defined by the adoption of initial underhand grasp postures. For final right end-down trials, end-state comfort was defined by the adoption of initial overhand grasp postures. Given that the grasp posture data did not meet the assumptions of parametric statistical tests (i.e., homogeneity and normal distribution), the proportion of trials in which end-state comfort was satisfied was determined for each participant, and then normalized using an arcsine transformation to transform the data prior to statistical analysis.

Statistical quantification of the differences in kinematic characteristics were conducted on the following dependent variables: bimodal grasp aperture profiles (proportion of total trials), reaction time (time period from initial stimulus presentation to start button release), reach-to-grasp time (time period from start button release to cradle switch release), reach-to-grasp phase peak velocity (highest point on the resultant reach-to-grasp phase velocity curve), reach-to-grasp phase time to peak velocity (relative time from reach-to-grasp phase movement onset to reach-to-grasp phase WJC peak velocity), grasp-to-place time (time period from cradle switch release to target disc activation), grasp-to-place phase peak velocity (highest point on the resultant grasp-to-place phase velocity curve). For each dependent variable, the average of each condition was submitted to a RM ANOVA with the factors trial type and final object end-orientation.

To determine grasp posture correction latency during the reach-to-grasp phase, we calculated the point in time where corrections in grasp posture were first initiated. Grasp posture correction latency for the final left end-down condition was defined as the point in time of minimum hand pronation/supination angle, whereas grasp posture correction latency for the final right end-down condition was defined as the point time of maximum hand pronation/supination. Relative and absolute grasp posture correction latency was defined as the time interval between reach-to-grasp onset to time to minimum/maximum of hand pronation/supination angle (depending on final object end-orientation condition).

Preliminary analyses were conducted to check for normality, sphericity (Mauchly test), univariate and multivariate outliers, with no serious violations noted. Results with p-values<0.05 were considered significant. Partial eta-squared (η^2^_p_) values were calculated for all *F*-tests as an indicator of effect size. Significant main effects and interactions were compared using Bonferroni corrected post-hoc analysis.

## Results

### Grasp posture and kinematic characteristics of non-perturbed trials

#### Grasp behavior

The proportions of trials in which initial grasp postures resulted in comfortable end postures for non-perturbed trials are displayed in Figure 3A. In line with previous work [5], participants overwhelmingly selected initial grasp postures that ensured comfort at the end of the movement (mean end-state comfort compliance = 98%). Paired samples t-test indicated that the proportion of trials in which end-state comfort was satisfied was similar for left end-down (96%) and right end down (99%) conditions, t(14) = −1.723, p = 0.107.

**Figure 3:**
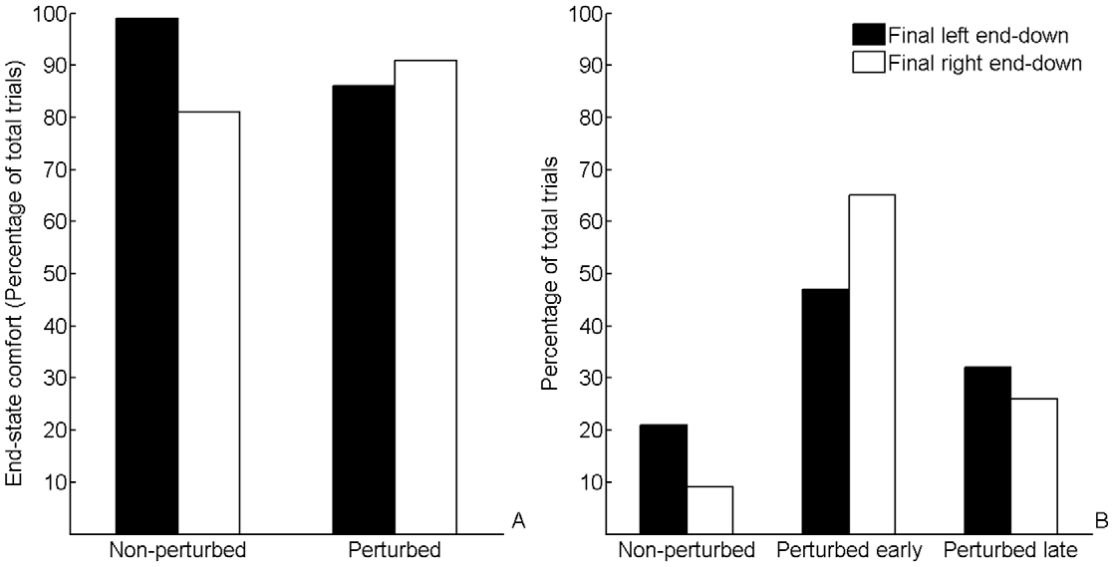
End-state comfort satisfaction and employment of correction strategy. (A) Proportions of trials in which initial grasp postures resulted in comfortable end postures (i.e., end-state comfort) for non-perturbed and perturbed trials. (B) Proportions of trials in which participants employed an early correction strategy, a late correction strategy, or did not change initial grasp posture (no-correction). Black bars refer to left end-down conditions. White bars refer to right end-down conditions.

#### Temporal and kinematic characteristics of non-perturbed trials

Representative relative reach-to-grasp phase grip aperture and hand orientation trajectories for non-perturbed trials are illustrated in Figure 4A. After start button release, participants immediately adjusted their hand pronation/supination movements so that the object could be grasped with an end-state comfort compliant grasp posture. The temporal characteristics of the reach-to-grasp and grasp-to-place phase were not influenced by the required final object end orientation. With respect to movement kinematics, stereotypical movement trajectories were observed for all participants. The WJC velocity profiles were bell-shaped, with an accelerative phase that was shorter than the decelerative phase during both the reach-to-grasp and the grasp-to-place phase of the movement (see Figure 5). On average, participants took 685 ms to grasp the object (reach-to-grasp time), and 773 ms to place the object into the target disc once the object was lifted from the cradle (grasp-to-place time). Average maximum grip aperture (MGA) was 138 mm, and occurred at 75% of the reach-to-grasp phase (absolute time to MGA: 517 ms), (see Figure 6).

**Figure 4:**
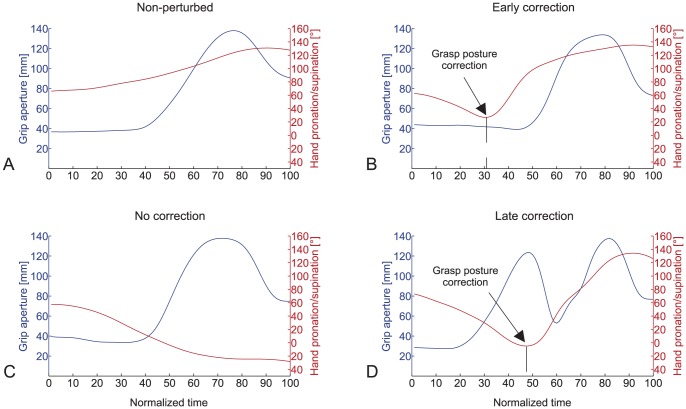
Representative grip aperture and hand orientation profiles for during the reach-to-grasp phase for A) non-perturbed trials, B) early correction perturbed trials, C) no-correction perturbed, and D) late correction perturbed trials. Corrections in grasp posture occurred at 30.1% of normalized reach-to-grasp time for early correction trials (panel C), and 45.9% of normalized reach-to-grasp time for late correction trials (panel D). Depicted are representative profiles for final left end-down trials. The pattern of grip aperture and hand orientation profiles are similar to final right end-down trials, with the exception that the hand orientation became more pronated throughout the reach-to-grasp phase.

**Figure 5:**
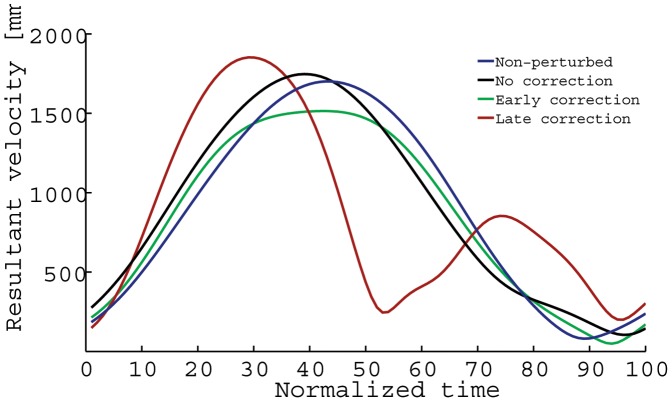
Representative relative velocity profiles during the reach-to-grasp phase.

**Figure 6:**
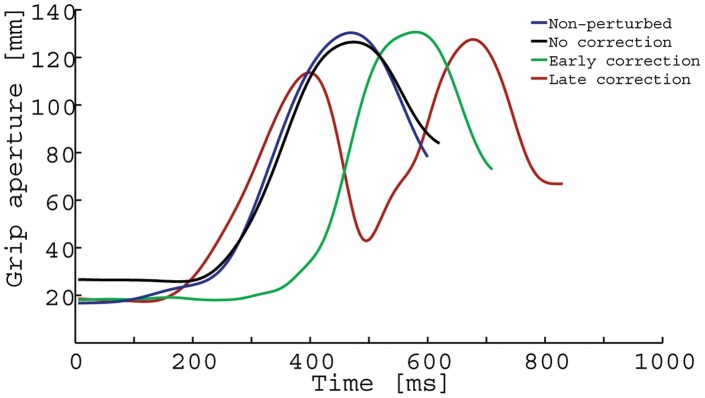
Representative absolute maximum grip aperture profiles during the reach-to-grasp phase.

### Grasp posture and kinematic characteristics of perturbed trials

#### Grasp behavior

Participants did not change grasp posture in 15% of perturbed trials (*no correction*). However, closer inspection of the video data revealed that participants corrected their grasp postures in 85% of trials using one of two strategies, which did not change across the experimental session, and were not due to individual differences. In 56% of perturbed trials, the formation of the appropriate grasp posture was more subtle, and did not include any overt changes in grasp posture (overhand vs. underhand). *Early corrections* in grasp posture were identified kinematically by unimodal reach-to-grasp phase grip aperture profiles and <125° change in the pro- supination angle. There were also a number of perturbed trials (29%) where participants would approach the object with one grasp posture, and then suddenly change grasp posture (e.g., approaching with an underhand grasp posture then switching to an overhand grasp posture). *Late corrections* in grasp posture were identified kinematically by biphasic approach phase grasp aperture profiles, with >125° change in the pro- supination angle.

#### Corrective Strategy

Proportions of early correction, late correction, or no-correction trials for left end-down and right end-down conditions are depicted in Figure 3B. A RM ANOVA on the factors corrective strategy (no correction, early correction, late correction) and final object end-orientation (left end down, right end down) revealed a significant main effect of corrective strategy, F(2,28) = 6.847, p = 0.004, η^2^_p_ = 0.328. Post-hoc analysis indicated that the percentage of trials in which participants did not change grasp posture (perturbed no-correction = 15%) was significantly lower (p = 0.007) than the percentage of trials in which participants made early corrections in grasp posture (56%). The difference between early correction and late correction trials (29%), and between perturbed no-correction and late correction trials was not significant (all p's>0.05). Thus, participants were more likely to correct their grasp postures early in the reach-to-grasp phase when the intended object end-orientation had changed.

The interaction between corrective strategy and final object end-orientation was significant, F(2,28) = 3.62, p = 0.040, η^2^_p_ = 0.206. Although post hoc tests indicated that there were no differences in corrective strategy for the final left end-down condition (perturbed no-correction = 21%, perturbed early = 47%, perturbed late = 32%), it did reveal that the proportion of trials in which participants used an early correction strategy (65%) for the final right end-down condition was significantly higher than the percentage of trials in which participants did not change grasp posture (9%), p<0.001. The difference between early correction and late correction trials (65% vs. 26%), and between perturbed no-correction and late correction trials (9% vs. 26%) for the final right end-down condition was not significant (p>0.05).

#### Temporal and kinematic characteristics of perturbed trials

Although the temporal and kinematic characteristics of perturbed no-correction trials were similar to non-perturbed trials (Figure 4C), perturbed early and late grasp posture correction trials differed from non-perturbed trials in a number of ways. Figure 4B and 4D depict representative reach-to-grasp phase grip aperture and hand orientation trajectories for early correction perturbed trials, and late correction perturbed trials. During early correction trials, hand pronation/supination angles were initially biased toward a grasp posture that would satisfy end-state comfort (based on end-orientation of the initial stimulus), and then 239 ms (30.1% of normalized reach-to-grasp time) after start button release, participants adjusted their hand pronation/supination movements so that the object could be grasped with an end-state comfort compliant grasp posture. The velocity profiles were similar to the unperturbed trials but peak velocity values were somewhat smaller (see Figure 5). Furthermore, participants took 786 ms to grasp the object (reach-to-grasp time), and 812 ms to place the object into the target disc once the object was lifted from the cradle (grasp-to-place time). Early corrections in grasp posture formation featured unimodal grasp aperture profiles, with MGA values (137 mm) occurring 78% into the reach-to-grasp phase (614 ms, see Figure 6).

During late correction trials (see Figure 4D), participants' pronation/supination angles were initially strongly biased toward a grasp posture that would satisfy end-state comfort (based on end-orientation of the initial stimulus), and then after approximately 399 ms (45.9% reach-to-grasp time) corrected their movements by pronating or supinating the hand (depending on condition) to form the necessary end-state comfort compliant grasp posture. These corrections in grasp posture were accompanied by bimodal velocity and grasp aperture profiles in 91% of trials (see Figures 5 and 6). The first MGA peak (111 mm) occurred early in the movement (relative time to MGA: 46%, absolute time to MGA: 393 ms), which was followed by a closing of the hand. The hand then gradually re-opened to reach a second MGA peak (138 mm) at 84% of reach-to-grasp phase (absolute time to MGA: 716 ms). Average reach-to-grasp times and grasp-to-place times were 865 ms and 800 ms, respectively.

A RM ANOVA (2×2) revealed a significant main effect of corrective strategy on relative grasp posture correction latency, with significantly shorter grasp posture correction latencies for early correction (30.1%) compared to late correction trials (45.9%), F(1,14) = 87.534, p<0.001, η^2^_p_ = 0.862. Differences in the absolute grasp posture correction latency also revealed a significant main effect of corrective strategy, F(1,14) = 134.089, p<0.001, η^2^_p_ = 0.905. Grasp posture correction latencies occurred after 239 ms for early correction trials and 398 ms for late correction trials. Relative and absolute grasp posture correction latencies was not influenced by final object end-orientation (respectively, F(1,14) = 1.483, p = 0.243, η^2^_p_ = 0.096 and F(1,14) = 0.091, p = 0.767, η^2^_p_ = 0.006).

### Comparison of non-perturbed and perturbed trials

#### Grasp behavior

The proportion of trials in which end-state comfort was satisfied was higher for non-perturbed (97%) compared to perturbed trials (85%), and for final right end-down (95%) compared to final left end-down conditions (84%). However these effects failed to reach statistical significance [respectively, F(1,14) = 4.33, p = 0.056, η^2^_p_ = 0.236 and F(1,14) = 4.45, p = 0.053, η^2^_p_ = 0.241].

#### Temporal and kinematic characteristics

The proportion of trials with bimodal grasp aperture profiles was significantly larger for perturbed late (91%), compared to perturbed early (7.5%) and non-perturbed trials (2%), F(2,28) = 181.506, p<0.001, η^2^_p_ = 0.928. The difference between perturbed early and non-perturbed trials did not reach significance (p = 0.350). The effect of final object end-orientation was not significant, F<1.0.

Average reaction time values were similar regardless of trial type (non-perturbed = 460 ms, perturbed early correction = 478 ms, perturbed late correction = 463 ms), F(2,28) = 0.912, p = 0.413, η^2^_p_ = 0.061. The main effect of final object end-orientation was also non-significant (final left end-down = 462 ms, final right end-down = 472 ms), F(1,14) = 0.310, p = 0.586, η^2^_p_ = 0.032. In short, reaction time values were remarkably homogenous regardless of upcoming task differences and correction strategy.

The manipulation of final object end-orientation had a significant effect on reach-to-grasp time, with shorter reach-to-grasp time values for final left end-down (763 ms), compared to final right end-down object end-orientation conditions (794 ms), F(1,14) = 5.099, p = 0.040, η^2^_p_ = 0.267. There was also a main effect of trial type on reach-to-grasp time, F(2,28) = 34.575, p<0.001, η^2^_p_ = 0.712. Bonferroni corrected post-hoc analysis revealed significant shorter average reach-to-grasp times for non-perturbed trials (685 ms) compared to both early correction (786 ms) and late correction trials (865 ms), and significantly shorter average reach-to-grasp times for early correction trials compared to late correction trials (both p's<0.001). The interaction between final object end-orientation and trial type was not significant (F<1.0).

There was a main effect of trial type on average reach-to-grasp peak velocity values, F(2,28) = 43.763, p<0.001, η^2^_p_ = 0.758. Post-hoc analysis revealed significantly higher peak velocity values for non-perturbed trials (1698 mm/s) and late correction trials (1702 mm/s) compared to early correction trials (1457 mm/s), p<0.001. Peak velocity values were similar for non-perturbed and late correction trials. The interaction between trial type and final object end-orientation was also significant, F(2,28) = 4.268, p = 0.024, η^2^_p_ = 0.234. Average peak velocity values were smaller for early correction final left end-down trials (1422 mm/s), compared to both non-perturbed final left end-down (1632 mm/s) and late correction final left end-down trials (1729 mm/s), p<0.001. Similarly, mean peak velocity was smaller for early correction trials (1492 mm/s) compared to non-perturbed (1764 mm/s) and late correction trials (1676 mm/s) for the final right end-down condition p<0.001. In sum, mean peak velocity was lower for early correction trials (compared to non-perturbed and late correction trials) for final left and final right end-down conditions.

Average reach-to-grasp time to peak velocity was influenced by trial type F(2,28) = 55.248, p<0.001, η^2^_p_ = 0.798, with shorter average time to peak velocity values for late correction trials (27.1%) compared to non-perturbed (42.6%) and early correction trials (40.4%), p<0.001. The interaction between trial type and final end-orientation was also significant, F(2,28) = 5.840, p = 0.008, η^2^_p_ = 0.294. Although average time to peak velocity was similar for final left and final right end-down conditions for early correction (respectively, 39.3% and 41.5%) and late correction trials (respectively, 27.2% and 27.1%), time to peak velocity values were significantly larger for final left end-down conditions for non-perturbed trials (final left end-down = 44.5%, final right end-down = 40.7%), p<0.001.

On average, grasp-to-place time values were similar irrespective of trial type (non-perturbed = 773 ms, perturbed early correction = 812 ms, perturbed late correction = 800 ms), F(2,28) = 1.890, p = 0.170, η^2^_p_ = 0.119. The influence of final object end-orientation was also non-significant, F(1,14) = 6.677, p = 0.22, η^2^_p_ = 0.323, with similar grasp-to-place times for final left and final right end-down conditions (817 ms and 773 ms respectively). Average peak velocity values were similar irrespective of trial type (non-perturbed = 704 mm/s, perturbed early = 696 mm/s, perturbed late = 714 mm/s), F(2,28) = 0.993, p = 0.383, η^2^_p_ = 0.066. The effects of final object end-orientation were also non-significant (final left end-down = 691 mm/s, final right end-down = 718 mm/s), F(1,14) = 3.134, p = 0.098, η^2^_p_ = 0. 183.

## Discussion

The purpose of this study was to examine how modifications in action goals influence grasp posture planning during a unimanual grasping and placing task. Congruent with previous research [8]–[9], participants overwhelmingly selected initial grasp postures that ensured comfortable hand postures at the end of the movement during non-perturbed trials. Non-perturbed reach-to-grasp trajectories were smooth and bell-shaped, with the formation of end-state compliant grasp postures starting at reach-to-grasp onset that did not feature any corrections in grasp posture or deviations in hand pronation/supination. Taken together, these results support the notion that goal postures are represented and planned prior to movement execution [8]–[9], [20]. If individuals were planning their postures while reaching for the object, we should have observed neutral hand pronation/supination angles during the early reach-to-grasp phase, with the formation of end-state compliant grasp postures occurring later in time. That grasp posture formation started at the beginning of the reach-to-grasp phase indicates that participants had already selected the appropriate end-state comfort compliant grasp posture prior to movement execution.

Congruent with recent studies indicating that reach-to-grasp hand [2], [5]–[7], [21] and finger [21]–[22] kinematics are affected by the action end-goals of a task, the grasp posture and kinematic data from perturbed trials demonstrate that initial grasp postures are highly influenced by the action end-goals of the task (i.e., required final end-orientation of the object), such that participants will modify their reach-to-grasp movements when faced with an unexpected change in action goal. That is, when the secondary stimulus indicated that the action goal (i.e., required object end orientation) had changed, participants switched from an overhand to underhand grasp posture during final left end-down trials, and from an underhand to an overhand grasp posture during final right end-down trials, in 85% of the trials. The modification of grasp postures in response to action goal perturbation is not a trivial one, as participants were able to complete the task using either an overhand or an underhand grasp posture. Further, grasp posture plans were modified so that they ensured comfortable postures at the end of the movement, indicating that end-state comfort is a highly prioritized planning constraint during unimanual object manipulation tasks.

Modifications in grasp posture occurred early or late in the reach-to-grasp phase (30% and 46% of normalized reach-to-grasp time, respectively), and were characterized by longer reach-to-grasp times, and shorter time to peak velocity values (indicative of a longer overall deceleration phase) compared to non-perturbed trials. Of the two grasp posture correction strategies, participants were more likely to initiate grasp posture corrections early in the reach-to-grasp movement. During these early correction trials, initial grasp formation was biased toward a grasp posture that would satisfy end-state comfort (with respect to the initial stimulus), and then 239 ms after reach-to-grasp onset grasp postures were corrected so that the object could be grasped with an end-state comfort compliant grasp posture. Similar to non-perturbed trials, grip aperture profiles for early correction trials were unimodal, with maximum grip aperture occurring at 78% of reach-to-grasp phase. From the analysis of grasp posture correction latency during early correction trials we were able to infer that the minimal amount of time required to process errors in grasp posture and initiate the appropriate correction response to be approximately 239 ms. The minimal correction time values for perturbed trials are similar to those reported in studies of reaching movements to perturbed object size [12], [23]–[25], and greater than reported for visual closed-loop feedback [26].

In comparison to early corrections in grasp posture, late corrections were more conspicuous in nature. Initial grasp formation was strongly biased toward the posture that would result in comfortable end postures for the initial stimuli. Then after approximately 398 ms, reach trajectory was adjusted to form the necessary end-state comfort compliant grasp posture. These corrections in grasp posture were accompanied by bimodal grasp aperture profiles. Similar to Paulignan et al. [14], we found that the first peak in grasp aperture occurred early in the movement (46%), then a closing of the hand, followed by a second peak in grasp aperture that occurs 84% into the reach-to-grasp phase. The amplitude of the first grasp aperture peak (111 mm) was smaller than the second late correction grasp aperture peak (138 mm), as well as the maximum grasp aperture during both non-perturbed (138 mm), and early correction (137 mm) trials.

Why did participants employ an early correction strategy in some trials, and a late correction strategy in other trials? One possibility is that participants selected the more time-inefficient late correction strategy at the start of the experimental session, and changed to a more effective early correction strategy as they learnt to compensate for the perturbation in action goal [27]–[28]. This hypothesis was not supported, as the selection of grasp posture correction strategy was relatively constant across the time course of perturbed trials. However, it is possible that the relatively low number of perturbed trials in the present study were not sufficient to cause a complete shift from the more time-inefficient late correction strategy to the more effective early correction strategy.

Alternatively, it is possible that participants focused more on speed during late correction trials compared to trials where an early correction strategy was employed. Support for this hypothesis can be derived from the kinematic data. First, the opening of the hand (grip aperture profile) was initiated earlier during late correction trials compared to early correction trials (see Figure 6). Second, peak velocity values of the reach-to-grasp phase were larger for late correction compared to early correction trials (see Figure 5). That is to say, the formation of an appropriate grasp (with respect to the initial stimulus) was already advanced until participants noticed and responded to the change in action goal (i.e., the secondary stimulus).

A third possibility is that the selection of an early or late correction strategy might arise from stimulus processing effects. During perturbed trials, the secondary visual stimulus depicted the required (changed) object end-orientation, whereas the auditory stimulus only indicated a change in object end-orientation. It is possible that uncertainty about the auditory stimulus mapping (i.e., whether the tone required a change in object end-orientation or not) participants may have used the visual stimulus to confirm whether a change in action goal had occurred. The reliance on both stimulus sources may have delayed the grasp correction response, resulting in a late correction strategy. In contrast, if the auditory stimulus mapping was clear, they did not have to use the visual stimuli to confirm whether a change in action goal had occurred. In this situation, participants were able to more quickly decide whether the action goal had been perturbed, resulting in the ability to correct grasp posture early in the reach-to-grasp phase (early correction strategy).

In general, studies on arm trajectory modifications during reaching have reported that changes in object size and location result in a bimodal pattern of grip aperture [11]–[13], and is often regarded as evidence that the original motor plan is cancelled and substituted by an updated movement plan, or that a corrective motor plan is superimposed on top of the originally planned one [29]. In the “abort-replan” strategy, the original motor plan specifying the motion from the hand position at the start of the trial and the hand position at the target location (i.e., intended action goal) is aborted in response to a perturbation, and a new motor plan (specifying movement from the current position to the final hand position) is conceived. In contrast, the “superimposition” strategy maintains that modifications in reaching movements involve the vectorial summation of two independent trajectory plans, with the first plan specifying motion between the original hand position and the target location (i.e., intended action goal), and the second, time-shifted plan specifying motion between the hand position at the time of the perturbation and at the position of the new action goal.

In the current task, participants had to choose between two distinct grasp postures (overhand vs. underhand). Superimposing a new grasp posture plan on top of the originally planned one would result in either a “thumb up” or “thumb down” grasp posture. Given that the object could not be grasped when the hand was oriented in a thumb up or down posture, it is unlikely that corrections in grasp posture reflect a modification in the existing plan. Rather, we hypothesize that corrections in grasp posture are accomplished by canceling the original plan and completely replanning the grasp posture plan. That said, the replanning of grasp postures does not come without costs. In line with previous studies in which target position [14], [17], object size [11]–[12], and object orientation are perturbed [21], we observed longer reach-to-grasp times, which were accompanied by a lengthening of the deceleration phase, for perturbed trials relative to non-perturbed trials. These kinematic differences indicate that there are motor control costs associated with grasp posture corrections, and that these corrections cannot occur within the initially prescribed movement duration.

Based on the results of the present study, we hypothesize that grasp posture planning is controlled via both feedforward and feedback mechanisms [6]–[7]. Prior to movement execution, feedforward control mechanisms are responsible for grasp posture planning. These feedforward mechanisms consist of an internally generated or predictive model of the movement, and incorporate overarching action goals (e.g., grasping an empty cup vs. grasping a cup filled with hot coffee), and specific factors relating to the object (e.g., object fragility, affordance), target (e.g., precision requirements, location), and the individual (e.g., sensitivity for comfortable final postures, compromised cognitive abilities). Feedforward models allow the CNS to predict the sensory consequences associated with self-generated movement to estimate the current state (e.g., position, velocity, and orientation) of the reaching limb, and compare this estimate with the actual sensory feedback [30]. Any errors in these predictions are used to issue corrective commands to the limbs. Grasp postures are also controlled via feedback mechanisms, in which proprioceptive and environmental visual cues are continuously processed to supervise the ongoing movement [31]. When an unanticipated external event occurs (e.g., change in action goal, presence of an obstacle), a command signal is generated and used to replan grasp postures. Consistent with this hypothesis, perturbations in action goal resulted in grasp posture corrections that occurred at 239 ms for early correction trials, and grasp posture corrections that occurred at 399 ms with bimodal grip aperture profiles for late correction trials.

In sum, the results of the present study indicate that individuals are able to make grasp posture corrections when an unanticipated event occurs at, or shortly after, reach onset. When the action goal of the task unexpectedly changed, individuals modified their grasp posture plans to ensure comfortable postures at the end of the movement, indicating that end-state comfort is a major determinant in grasp posture planning during unimanual object manipulation tasks. Corrections in grasp posture occurred early or late in the reach-to-grasp phase, and provide direct evidence that human prehension movements are controlled by feedforward and feedback mechanisms.
